# The impact of immunoglobulin G N-glycosylation level on COVID-19 outcome: evidence from a Mendelian randomization study

**DOI:** 10.3389/fimmu.2023.1217444

**Published:** 2023-08-18

**Authors:** Feiwu Long, Chenghan Xiao, Huijie Cui, Wei Wang, Zongze Jiang, Mingshuang Tang, Wenqiang Zhang, Yunjie Liu, Rong Xiang, Li Zhang, Xunying Zhao, Chao Yang, Peijing Yan, Xueyao Wu, Yutong Wang, Yanqiu Zhou, Ran Lu, Yulin Chen, Jiayuan Li, Xia Jiang, Chuanwen Fan, Ben Zhang

**Affiliations:** ^1^ Department of Gastrointestinal, Bariatric and Metabolic Surgery, West China-PUMC C.C. Chen Institute of Health, West China School of Public Health and West China Fourth Hospital, Sichuan University, Chengdu, China; ^2^ Department of Maternal, Child and Adolescent Health, West China School of Public Health and West China Fourth Hospital, Sichuan University, Chengdu, Sichuan, China; ^3^ Department of Epidemiology and Biostatistics, Institute of Systems Epidemiology, West China-PUMC C. C. Chen Institute of Health, West China School of Public Health and West China Fourth Hospital, Sichuan University, Chengdu, China; ^4^ Department of Urology and Pelvic Surgery, West China-PUMC C. C. Chen Institute of Health, West China School of Public Health, and West China Fourth Hospital, Sichuan University, Chengdu, China; ^5^ Laboratory of Stem Cell Biology, West China Hospital, West China School of Medicine, Sichuan University, Chengdu, China; ^6^ Department of Environment and Occupational Health, West China School of Public Health and West China Fourth Hospital, Sichuan University, Chengdu, China; ^7^ Department of Nutrition and Food Hygiene, West China-PUMC C.C. Chen Institute of Health, West China School of Public Health and West China Fourth Hospital, Sichuan University, Chengdu, Sichuan, China; ^8^ Department of Oncology, Linköping University, Linköping, Sweden; ^9^ Department of Biomedical and Clinical Sciences, Linköping University, Linköping, Sweden

**Keywords:** Mendelian randomization, COVID‐19, IgG N-glycosylation, causality, inflammation

## Abstract

**Background:**

The coronavirus disease 2019 (COVID-19) pandemic has exerted a profound influence on humans. Increasing evidence shows that immune response is crucial in influencing the risk of infection and disease severity. Observational studies suggest an association between COVID‐19 and immunoglobulin G (IgG) N-glycosylation traits, but the causal relevance of these traits in COVID-19 susceptibility and severity remains controversial.

**Methods:**

We conducted a two-sample Mendelian randomization (MR) analysis to explore the causal association between 77 IgG N-glycosylation traits and COVID-19 susceptibility, hospitalization, and severity using summary-level data from genome-wide association studies (GWAS) and applying multiple methods including inverse-variance weighting (IVW), MR Egger, and weighted median. We also used Cochran’s Q statistic and leave-one-out analysis to detect heterogeneity across each single nucleotide polymorphism (SNP). Additionally, we used the MR-Egger intercept test, MR-PRESSO global test, and PhenoScanner tool to detect and remove SNPs with horizontal pleiotropy and to ensure the reliability of our results.

**Results:**

We found significant causal associations between genetically predicted IgG N-glycosylation traits and COVID-19 susceptibility, hospitalization, and severity. Specifically, we observed reduced risk of COVID-19 with the genetically predicted increased IgG N-glycan trait IGP45 (OR = 0.95, 95% CI = 0.92–0.98; FDR = 0.019). IGP22 and IGP30 were associated with a higher risk of COVID-19 hospitalization and severity. Two (IGP2 and IGP77) and five (IGP10, IGP14, IGP34, IGP36, and IGP50) IgG N-glycosylation traits were causally associated with a decreased risk of COVID-19 hospitalization and severity, respectively. Sensitivity analyses did not identify any horizontal pleiotropy.

**Conclusions:**

Our study provides evidence that genetically elevated IgG N-glycosylation traits may have a causal effect on diverse COVID-19 outcomes. Our findings have potential implications for developing targeted interventions to improve COVID-19 outcomes by modulating IgG N-glycosylation levels.

## 1 Introduction

The severe acute respiratory syndrome coronavirus 2 (SARS-CoV-2) continues to evolve and spreads worldwide, causing over 600 million infections and approximately 6.5 million deaths as of September 2022 (1). Individuals infected with SARS-CoV-2 develop COVID-19 symptoms of varying severity, including asymptomatic infection, mild respiratory symptoms, respiratory failure, and death (2). Only a small proportion of infected individuals develop severe disease, suggesting the potential modulating role of some predisposing factors. Thus, investigating the mechanisms underlying the heterogeneity of infection and symptoms is crucial for developing efficient prevention and treatment of COVID-19. Several risk factors have been reported to increase the risk of COVID-19 severity and mortality, including older age, obesity, and chronic diseases such as diabetes, cardiovascular diseases, chronic kidney disease, and respiratory diseases (3, 4). Meanwhile, increasing evidence has shown that host genetic factors modulate the risk of infection and disease severity by affecting immune-related genes (4, 5). In particular, the cytokine storm and overactivation of the immune system are closely related to the severity of COVID-19 (6), suggesting the critical role of an effective host immune response and the devastating effect of immune dysregulation (7).

Immunoglobulin G (IgG), as the prevalent isotype of antibodies, plays a pivotal role in humoral immunity against pathogens, as it can mediate immune responses to viral infections and participate in systemic antiviral immunity and inflammation response (8). IgG mediates immune response through interactions of its fragment crystallizable (Fc)-Fc gamma receptor (FcγR) and Fc-complement 1q (9). The functional diversity of IgG is mediated by the heterogeneous glycosylation of the Fc domain Asn297, which carries complex N-glycans (10). Recent observational studies have indicated the association of IgG N-glycosylation with COVID-19 severity (11, 12). The levels of bisecting N-acetylglucosamine (GlcNAc) and galactosylation of IgG were negatively related to COVID-19 severity (13). Additionally, the fucosylation and sialylation of IgG were observed to be positively correlated with COVID-19 severity, which closely contributed to the ADCC-regulated enhancement of inflammatory cytokines (14, 15). Moreover, previous studies also found that decreased fucosylation, galactosylation, and sialylation of anti-SARS-CoV-2 IgG1, as well as high bisection were early inflammatory signals promoting more severe disease in COVID-19 patients (16–18).

Although observational studies demonstrate that changes in IgG glycome composition are closely related to the regulation of different immune processes (either pro-inflammation or anti-inflammation) in SARS-CoV-2 infection, uncertainties persist about whether these associations indicate a causal effect between IgG N-glycosylation and severity of COVID-19. Mendelian randomization (MR) study, which employs genetic variants that are randomly inherited from parents to offspring at conception, can help reduce confounding and eliminate reverse causation, often found in observational studies. Therefore, this study used MR to examine the potential causal relationship between IgG N-glycosylation traits and COVID-19 susceptibility, hospitalization, and severity based on publicly available GWAS summary statistics.

## 2 Methods

### 2.1 Data sources for IgG N-glycosylation

The summary-level GWAS data correlated with IgG N-glycosylation traits were obtained from the largest meta-analysis of GWAS on 8090 individuals of European ancestry (19). GWAS summary statistics were available for 77 IgG N-glycosylation traits (IGP1-77), including 23 directly measured N-glycosylation traits (IGP1-23)—determined by ultraperformance liquid chromatography (UPLC)—and 54 derived N-glycosylation traits (IGP24-77). Each UPLC peak primarily represents a major diantennary complex N-glycan structure in the directly measured traits. These structures may exhibit features such as core-fucose, bisecting N-acetylglucosamine (GlcNAc), terminal galactose, and terminal sialic acids on the antennae. Conversely, the derived N-glycosylation traits describe the abundances or ratios of specific groups of glycans that share certain structural characteristics (**Additional File 1; Table S1**).

### 2.2 Data sources for susceptibility, hospitalization, and severity of COVID-19

In our study, we extracted summary statistics from the COVID-19 Host Genetics Initiative (www.covid19hg.org), focusing on the most recent dataset available at the time of our analysis (Round 7, April 2022). This data included three distinct COVID-19 phenotypes observed within individuals of European ancestry: COVID-19 susceptibility, COVID-19 hospitalization, and COVID-19 severity. COVID-19 susceptibility included any individuals diagnosed or self-reported as COVID-19 positive (including 1,226,616 cases and 2,475,240 population controls). COVID-19 hospitalization included individuals hospitalized for SARS-CoV-2 infection or COVID-19-related symptoms, excluding those who needed respiratory support or experienced fatal outcomes (including 32,519 cases and 2,062,805 population controls). Lastly, COVID-19 severity referred to cases that required respiratory support or resulted in death (including 13,769 cases and 1,072,442 population controls). Unfortunately, the dataset does not provide summary statistics for comparisons between two specific groups: the individuals who were hospitalized versus those who tested positive for SARS-CoV-2 but did not require hospitalization and those who were hospitalized versus those who had severe COVID-19 symptoms.

### 2.3 Choice of IgG N-glycosylation genetic instruments

**Figure 1:**
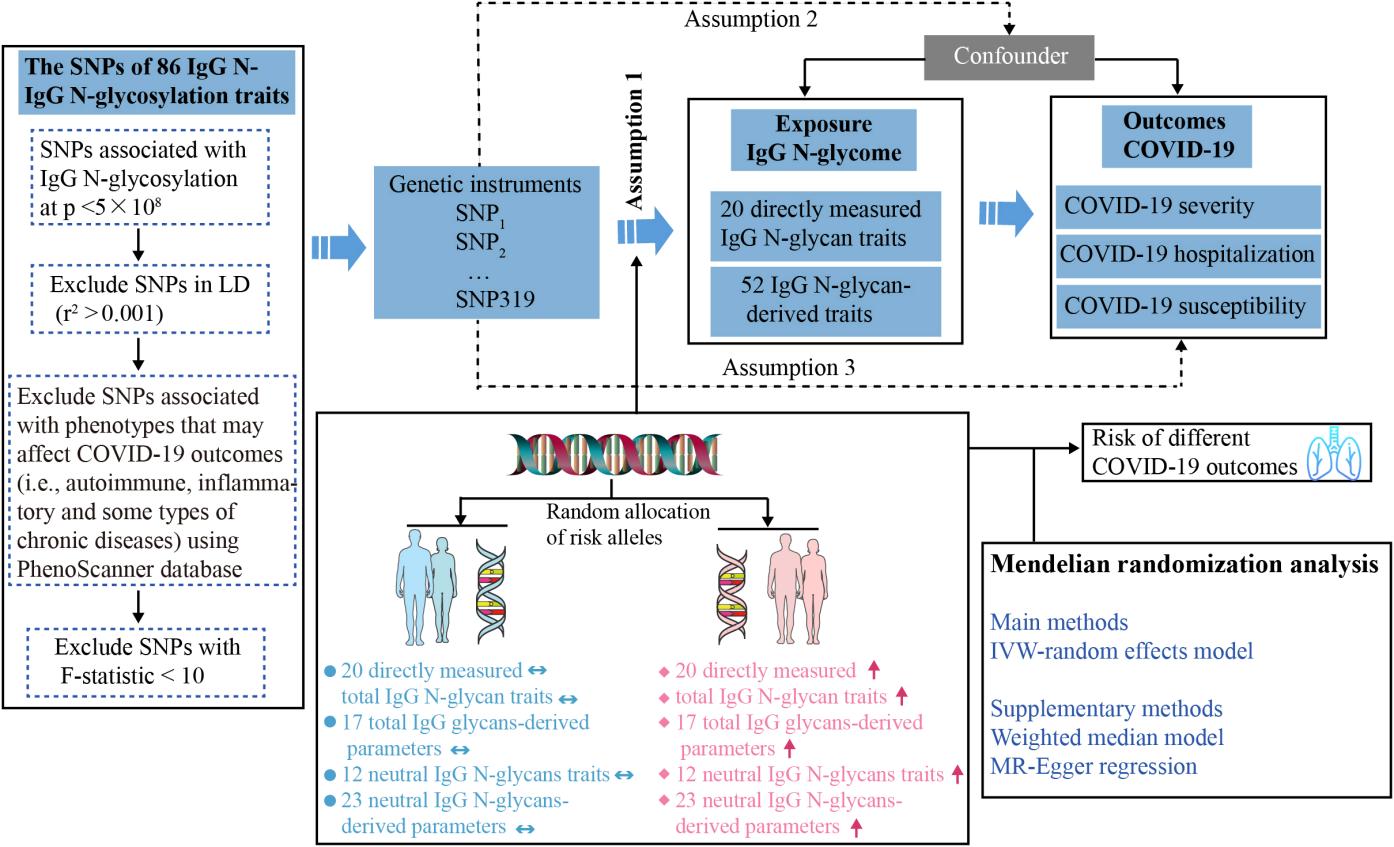
Study design overview and assumptions of the Mendelian randomization (MR) framework. Assumption 1 requires that the genetic variants selected as instrumental variables should have a strong association with the IgG N-glycosylation traits. Assumption 2 requires that these genetic variants should not be associated with any potential confounding factors. Assumption 3 requires that the selected genetic variants should have an effect on the COVID-19 outcome risk solely through the IgG N-glycosylation traits, rather than through alternative factors. Horizontal double-headed blue arrow represents the population unaffected by SNPs, and red up arrow denotes the population possessing high-risk SNPs. IgG N-Glycosylation, Immunoglobulin G N-Glycosylation; SNPs, single-nucleotide polymorphisms; LD, linkage disequilibrium; IVW, inverse-variance weighted.

To establish the genetic instruments for IgG N-glycosylation levels, we extracted genetic variants robustly associated with IgG N-glycosylation (**Figure 1**). SNPs associated with IgG N-glycosylation traits that reached genome-wide significance (p < 5×10^−8^) were selected as instrumental variables (IVs). We excluded SNPs in linkage disequilibrium (LD, r^2^ < 0.001 within a 10 Mb window). The R^2^ and F statistic of each SNP were calculated using the formulas: R^2^  =  2 × EAF × (1−EAF) × β^2^ and F statistic = R^2^ × (N−2)/(1−R^2^). SNPs with F statistic < 10 were excluded to ensure the robustness of IV and minimize weak instrument bias (20). We further used the PhenoScanner database (21) (Version 2, http://www.phenoscanner.medschl.cam.ac.uk/) to detect potential SNPs associated with the selected ones, which may affect COVID-19 outcomes (i.e., autoimmune, inflammatory and some types of chronic diseases including asthma, rheumatoid arthritis, diabetes, obesity, etc.). Then, the retained SNPs were extracted from the outcome datasets. Finally, 63 IgG N-glycosylation traits, each having at least two IVs, were included in this study (**Additional File 1; Table S2**).

### 2.4 Two-sample MR analysis

Three two-sample MR methods, including random-effect inverse-variance weighted (IVW), MR Egger, and weighted median, were performed using the “TwoSampleMR” package in R software (version 4.2.0) (22). The results of the IVW method were considered the major outcome, and the MR-Egger and weighted median were used to improve the IVW estimates as they can provide more robust estimates in a broader set of scenarios, despite being less efficient [i.e., wider confidence intervals (CIs)]. All results were presented as odds ratios (ORs) and corresponding 95% CIs of the COVID-19 outcomes per genetically predicted increase in IgG N-glycosylation level. We performed false discovery rate (FDR) correction for multiple testing using the Benjamini-Hochberg method to adjust the significance level thresholds, with significance defined as FDR < 0.05.

### 2.5 Sensitivity analysis

The Cochran’s Q statistic and leave-one-out analysis were performed to evaluate the degree of heterogeneity across SNPs. The MR-Egger intercept test and MR-PRESSO global test were conducted to detect horizontal pleiotropy. All analyses were conducted using the TwoSampleMR (version 0.5.6) and MRPRESSO (version 1.0) packages in R software (version 4.2.0). A P value < 0.05 indicated that the IVW results might be invalid due to horizontal pleiotropy.

## 3 Results

### 3.1 Choice of IgG N-glycosylation genetic instruments

To test for the causal effect of IgG N-glycosylation traits on the susceptibility and severity of COVID-19, we used genetic variants robustly associated with IgG N-glycosylation traits from the largest available meta-analysis of GWAS (**Table 1**). There were 77 IgG N-glycosylation traits which either directly measured abundances of individual N-glycans (glycan traits IGP1-23) or abundances of various N-glycans sharing similar structural characteristics and their ratios (glycan traits IGP 24-77) (19). The 319 independent SNPs for 64 IgG N-glycosylation traits were included in the current analysis after excluding LD variants and variants associated (at a genome-wide significant threshold of p < 5 × 10^−8^) with phenotypes that may affect COVID-19 outcomes (i.e., autoimmune, inflammatory, and some types of chronic diseases) using the PhenoScanner tool (**Figure 1**). The maximum number of IVs for the IgG N-glycosylation trait was eight, including IGP63, IGP68, IGP69, IGP74, IGP75, and IGP76. The mean and median number of IVs per IgG N-glycosylation trait in this set of 63 traits were 4.9 and 5, respectively. The F statistic for the remaining SNPs selected as IVs ranged from 33–1480, indicating a strong IV (23) (**Additional File 1; Table S3**).

**Table 1:** Sources of data for the analysis.

Phenotype	Source of genetic variants	
	Cohort	Participants
IgG N-glycosylation traits	Exposure	Meta-analysis of 77 IgG N-glycosylation traits GWAS on four cohorts of European descent (N = 8090).
COVID-19 susceptibility	Susceptibility	Meta-analysis of 22 GWASs performed in individuals of European ancestry: • Cases: 38,984 individuals with COVID-19 by laboratory confirmation, chart review, or self-report • Controls: 1,644,784 individuals without confirmation or history of COVID-19
COVID-19 severity	Hospitalized	Meta-analysis of 12 GWASs performed in individuals of European ancestry: • Cases: 9,986 hospitalized individuals with COVID-19 • Controls: 1,877,672 individuals without confirmation or history of COVID-19
	Severe disease	Meta-analysis of 15 GWASs performed in individuals of European ancestry: • Cases: 5,101 SARS-CoV-2-infected hospitalized individuals who died or required respiratory support (intubation, CPAP, BiPAP, continuous external negative pressure, or high-flow nasal cannula) • Controls: 1,383,241 individuals without confirmation or history of COVID-19

### 3.2 Causal effect of IgG N-glycosylation traits and COVID-19 susceptibility

**Figure 2:**
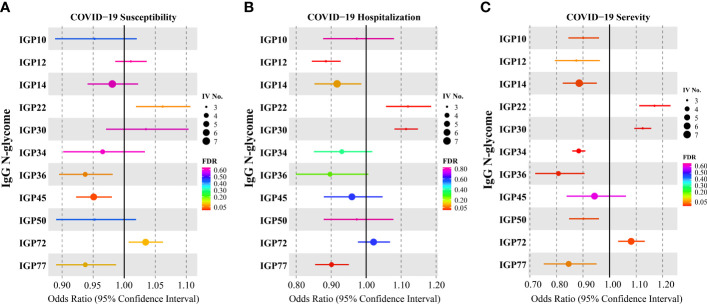
Causal effects for IgG N-glycosylation traits on COVID-19 outcomes. **(A)** Results of the Mendelian randomization (MR) analysis derived from the inverse-variance weighted (IVW) testing the effect of genetically determined IgG N-glycosylation traits on **(A)** COVID-19 susceptibility, hospitalization **(B)** and severity **(C)**.

In terms of COVID-19 susceptibility, we observed a significant causal association between genetically predicted increased IgG N-glycan trait IGP45 (The percentage of IGP6 glycan in total neutral IgG glycans, **Additional File 1; Table S1**) and COVID-19 susceptibility. Each standard deviation (SD) increase in genetically determined IGP45 was associated with a 5% decreased risk of COVID-19 infection (**Figure 2A**, **Additional File 1; Table S4**, OR = 0.95, 95% CI = 0.92–0.98; *FDR* = 0.019). Nevertheless, IGP45 was not associated with COVID-19 hospitalization (**Figure 2B**, **Additional File 1; Table S5**, OR = 0.96; 95%CI = 0.88–1.05, *FDR* = 0.601) or severity (**Figure 2C**, **Additional File 1; Table S6**, OR = 0.94, 95%CI = 0.84–1.06; *FDR* = 0.601). Except for the suggestive signals indicating a higher risk of COVID-19 infected with increased IGP22 (OR = 1.06, 95%CI = 1.02–1.11; *FDR* = 0.059) and IGP72 (OR = 1.03, 95%CI = 1.01–1.06; *FDR* = 0.088) traits, no causal relationship was observed with the rest of the 60 IgG N-glycan traits (**Additional File 1; Table S4**; *FDR* > 0.05). There was no evidence of SNP heterogeneity based on Cochran’s Q test and leave-one-out analysis (**Additional File 2; Tables S1 and S2**). The MR-Egger intercepts (**Additional File 2; Table S3**) and MR-PRESSO (**Additional File 2; Table S4**, MR-Egger intercept) showed no evidence of horizontal pleiotropy.

### 3.3 Causal effect of IgG N-glycosylation traits and COVID-19 hospitalization

Regarding COVID-19 hospitalization, after multiple testing corrections, two IgG N-glycosylation traits were causally associated with an increased risk of COVID-19 hospitalization. These traits included a directly measured IgG N-glycans, IGP22, and a total IgG N-glycans-derived trait, IGP30, which indicates the percentage of disialylation of fucosylated digalactosylated structures in total IgG glycans. Each SD increase in genetically determined IGP22 and IGP30 was associated with a 12% (OR = 1.12; 95%CI = 1.06–1.19; *FDR* = 0.004) and 11% (OR = 1.11; 95%CI = 1.08–1.15; *FDR* = 1.80×10^−10^) increased risk of COVID-19 hospitalization (**Figure 2B**; **Additional File 1; Table S5**). Furthermore, two IgG N-glycosylation traits were causally associated with a decreased risk of COVID-19 hospitalization, including a directly measured IgG N-glycans, IGP2, and a total IgG N-glycans-derived trait, IGP77, which indicates a ratio of digalactosylated structures with bisecting GlcNAc and all fucosylated digalactosylated structures (+/- bisecting GlcNAc).

The Cochran’s Q test showed no heterogeneity between IVs with either IVW or MR-Egger regression (**Additional File 3; Table S1**). Leave-one-out sensitivity analysis showed no evidence that a single SNP influenced the overall effect on both IGP12 and IGP77 (**Additional File 3; Table S2**). MR-Egger intercepts (**Additional File 3; Table S3**) and MR PRESSO did not show significant horizontal pleiotropy (**Additional File 3; Table S4**).

### 3.4 Causal effect of IgG N-glycosylation traits and COVID-19 severity

The strongest causal associations were observed between IgG N-glycosylation traits and COVID-19 severity, compared to those between IgG N-glycosylation traits and COVID-19 susceptibility or hospitalization. Our results (**Figure 2C**; **Additional File 1; Table S6**) indicated that IGP22 (OR = 1.17; 95%CI = 1.11–1.23; *FDR* = 6.22×10^−08^), IGP30 (OR = 1.12; 95%CI=1.09–1.16; *FDR*=6.94×10^−14^), and IGP72 (OR = 1.08; 95%CI = 1.03–1.13; *FDR* = 0.019) were associated with risk of COVID-19 severity.

Notably, five IgG N-glycosylation traits (IGP10, IGP14, IGP34, IGP36, and IGP50) were associated with a reduced risk of COVID-19 severity (**Figure 2C**; **Additional File 1; Table S6**). Each SD increase in genetically determined IGP10 (OR = 0.90; 95%CI = 0.84–0.96; *FDR* = 0.021), IGP14 (OR = 0.89; 95%CI = 0.82–0.95; *FDR* = 0.019), IGP34 (OR = 0.88; 95%CI = 0.86–0.91; *FDR* = 5.04×10^−16^), IGP36 (OR = 0.81; 95%CI = 0.72–0.91; *FDR* = 0.006), and IGP50 (OR = 0.90; 95%CI = 0.85–0.96; *FDR* = 0.019) was associated with a decreased risk of COVID-19 severity but was not associated with COVID-19 hospitalization (**Figure 2B**; **Additional File 1; Table S5**; *FDR* > 0.05).

A series of sensitivity analyses were conducted to assess the robustness of our results. No notable heterogeneities and horizontal pleiotropies were observed on Cochran’s Q test (**Additional File 4; Table S1**), leave-one-out sensitivity analysis (**Additional File 4; Table S2**), MR-Egger intercepts (**Additional File 4; Table S3**), and MR PRESSO (**Additional File 4; Table S4**) for all SNPs of IgG N-glycosylation traits either increasing or decreasing the risk of COVID-19 severity.

## 4 Discussion

To our knowledge, this is the first MR study to investigate the causal associations of genetically predicted IgG N-glycans with COVID-19 susceptibility, hospitalization, and severity. We found that increased genetically predicted IgG N-glycan of IGP45 was associated with a lower risk of COVID-19 susceptibility. The protective role of IGP45 has not been reported in previous observational studies (11, 17), although the role of IGP45 in COVID-19 susceptibility was similar to that in inflammatory diseases. The proportion of IGP45 is lower in chronic inflammatory and autoimmune disease cases (24, 25), suggesting its potentially important role in boosting immune responses. Concerning COVID-19 hospitalization or severity, however, no causal effect was observed for IGP45, indicating that IGP45 might not affect disease progression after infection. In addition, we found that IgG N-glycosylation traits might exhibit functional heterogeneity before and after SARS-CoV-2 infection.

The fact that only a small proportion of individuals infected with SARS-CoV-2 develop severe COVID-19 indicates the presence of certain predisposing factors. We, therefore, further evaluated the correlation between IgG N-glycosylation traits and severity by hospitalization and disease severity. IGP22, which directly measures IgG N-glycans, and IGP30, which measures the percentage of disialylation of fucosylated digalactosylated structures in total IgG glycans, were positively associated with the risk of COVID-19 hospitalization and severity. Previous studies on IgG glycosylation demonstrated that IGP22 and IGP30 were significantly higher in COVID-19 cases (15). Nonetheless, recent findings indicate that a lack of or decrease in sialylation, whether in anti-SARS-CoV-2 spike protein-specific (anti-S) IgG1 (17, 18) or total IgG N-glycans, is protective against severe symptoms and cytokine storm in COVID-19 (18, 26). Such discrepancy might be because we examined a pre-existing IgG N-glycans pool, whereas the previous studies focused on glycosylation dynamics following COVID-19 infection. Additionally, we analyzed directly measured and derived IgG glycan traits, while most previous reports primarily analyzed the levels of glycosylation. Although the increase in IGP30 was not directly measured, it was evident from derivative properties that describe the sialylation of galactosylated fucosylated structures in total IgG glycans. The current study only observed an increase in IGP30 in severe COVID-19, suggesting that IgG sialylation may play a role in anti-inflammatory activity and COVID-19 severity. Therefore, it is important to not only focus on the level of IgG glycosylation but also to examine detailed correlations between IgG glycan traits and COVID-19. There is a need to further explore the role of IGP22 and IGP30 as inflammation modulators in COVID-19 and the causal relationship and consequences of their changes during COVID-19 in relation to inflammation.

Five IgG N-glycosylation traits, including two directly measured IgG N-glycans (IGP10 and IGP14) and three total IgG N-glycans-derived traits (IGP34, IGP36, and IGP50), were associated with reduced risk of COVID-19 severity, consistent with prior findings that total IgG fucosylation levels were lower in severe COVID-19 cases (15). The IGP34 trait measures the ratio of fucosylated (without bisecting GlcNAc) monosialylated and disialylated structures in total IgG glycans, while IGP36 measures the ratio of all fucosylated sialylated structures with and without bisecting GlcNAc. Previous evidence has demonstrated that reduced fucosylation of IgG, which targets antigens from the viral envelope of the dengue virus, is associated with disease exacerbation (27). Furthermore, patients with severe COVID-19 have been shown to exhibit higher levels of fucosylated anti-SARS-CoV-2 IgG compared to those with mild disease (17), suggesting that fucosylation of IgG may protect against severe COVID-19 to some extent. This might be explained by IgG lacking or decreasing core fucosylation can increase their binding affinity to the Fc receptor FcRIIIa, leading to enhanced antibody-dependent cellular cytotoxicity (17). This enhanced cytotoxicity may be associated with a “cytokine storm” and overactivation of the immune system, which have been suggested as key features of severe COVID-19 (6). One observational study reported that severe COVID-19 was associated with increased total IgG fucosylation (11), and other studies found that total IgG fucosylation remained relatively stable (14, 17, 28). Various speculations have been proposed to explain the glycan variations of IgG Fc among populations. An example of the variation in the effect is that the severity can differ significantly from one patient to another, and not all severe patients will show increased levels of pro-inflammatory cytokines and IgG glycosylation features (13). Thus, the balance between anti-inflammatory and pro-inflammatory responses in the lungs following SARS-CoV-2 infection may be influenced by IgG glycosylation, which contributes to the different clinical phenotypes observed in COVID-19 patients with mild versus severe symptoms. The reported variations in fucosylation levels among COVID-19 patients could be attributable to the distinct glycosylation profiles of antigen-specific (Anti-S) or total IgG analyzed in each study, as well as the dynamic changes in the presence of antigen-specific IgG during the disease. However, the dynamic immune response and specific glycosylation profile of antigen-specific IgGs might not always be detectable at the level of total IgG, depending on their concentration and the timing of sampling. Moreover, limited sample sizes in previous studies have made it difficult to evaluate variations in total IgG glycosylation profiles. In our study, we investigated the causality between glycosylation features and COVID-19 outcomes by directly measuring each IgG N-glycan and deriving traits from total IgG N-glycans. Previous studies, however, mainly focused on identifying the relationship between glycosylation groups and COVID-19 outcomes. Finally, differences in IgG fucosylation may also be due to the diverse molecular mechanisms involved in the immune response to SARS-CoV-2. Therefore, the mechanisms underlying glycoalterations in IgGs associated with SARS-CoV-2 infection require further investigation.

However, there are some limitations worth mentioning. Firstly, our study was limited to participants of European ancestry, which may restrict the generalizability of our findings to other populations, such as those of Asian and African ancestry. Secondly, as a population-based cross-sectional study, our findings cannot establish causal relationships or provide pathophysiological inferences. Basic experiments *in vivo* or *in vitro* are necessary to confirm the association of IgG N-glycan biomarkers with COVID-19 outcomes. Thirdly, due to the unavailability of individual data, we were unable to conduct stratified analyses or adjust for other covariates using the available summary statistics dataset.

## 5 Conclusions

Overall, the current study suggests that IgG N-glycosylation status is causally associated with COVID-19 outcomes, but more research is needed to fully understand the mechanisms underlying these associations. Our findings may have implications for developing interventions that target IgG N-glycosylation traits to improve COVID-19 outcomes.

## Data availability statement

The original contributions presented in the study are included in the article/**Supplementary Material**. Further inquiries can be directed to the corresponding authors.

## Ethics statement

The studies involving humans were approved by The Mendelian randomization analysis conducted in this study utilized summary data from previously conducted studies that had obtained written informed consent and ethics approval. As the analysis involved secondary use of summary data, no additional ethical permit was required. The studies were conducted in accordance with the local legislation and institutional requirements. The participants provided their written informed consent to participate in this study.

## Author contributions

FL and CF contributed to data analysis and drafted the manuscript. CF, XJ, and BZ were responsible for the study conception, design, and revision of the paper. CX, and XZ contributed to the collecting data. WZ, HC, RX, LZ, CY, RL, YC, and JL contributed to the interpretation of results. FL, CF, and XJ contributed to the study design. All authors contributed to the article and approved the submitted version.

## Supplementary material

Additional file 1 Supplementary tables for MR analysis. **Table S1**. Definition and brief descriptions of directly measured and derived IgG N-glycosylation traits. **Table S2**. The instrumental variables for IgG N-glycosylation traits. **Table S3**. Estimated variance explained by SNPs and F statistics. **Table S4**. Association of genetically predicted IgG N-glycosylation traits with the risk of COVID-19 susceptibility by different Mendelian randomization approaches. **Table S5**. Association of genetically predicted IgG N-glycosylation traits with the risk of COVID-19 hospitalization by different Mendelian randomization approaches. **Table S6.** Association of genetically predicted IgG N-glycosylation traits with the risk of COVID-19 severity by different Mendelian randomization approaches.

Additional file 2 Supplementary tables for sensitivity analysis in MR analysis of COVID-19 susceptibility. **Table S1.** The Cochran Test of heterogeneity for IgG N-glycosylation traits on COVID-19 susceptibility. **Table S2**. The leave-one-out analysis for IgG N-glycosylation traits on COVID-19 susceptibility. **Table S3**. The MR-Egger intercepts analysis for IgG N-glycosylation traits on COVID-19 susceptibility. **Table S4**. The MRPRESSO test for IgG N-glycosylation traits on COVID-19 susceptibility

Additional file 3 Supplementary tables for sensitivity analysis in MR analysis of COVID-19 hospitalization. **Table S1**. The Cochran Test of heterogeneity for IgG N-glycosylation traits on COVID-19 hospitalization. **Table S2**. The leave-one-out analysis for IgG N-glycosylation traits on COVID-19 hospitalization. **Table S3**. The MR-Egger intercepts analysis for IgG N-glycosylation traits on COVID-19 hospitalization. **Table S4**. The MRPRESSO test for IgG N-glycosylation traits on COVID-19 hospitalization.

Additional file 4 Supplementary tables for sensitivity analysis in MR analysis of COVID-19 severity. **Table S1**. The Cochran Test of heterogeneity for IgG N-glycosylation traits on COVID-19 severity. **Table S2**. The leave-one-out analysis for IgG N-glycosylation traits on COVID-19 severity. **Table S3**. The MR-Egger intercepts analysis for IgG N-glycosylation traits on COVID-19 severity. **Table S4**. The MRPRESSO test for IgG N-glycosylation traits on COVID-19 severity.
